# Model-driven analysis of experimentally determined growth phenotypes for 465 yeast gene deletion mutants under 16 different conditions

**DOI:** 10.1186/gb-2008-9-9-r140

**Published:** 2008-09-22

**Authors:** Evan S Snitkin, Aimée M Dudley, Daniel M Janse, Kaisheen Wong, George M Church, Daniel Segrè

**Affiliations:** 1 Bioinformatics graduate Program, Boston University, Boston, MA 02215, USA; 2 Institute for Systems Biology, Seattle, WA 98103, USA; 3 McKinsey & Company, London, SW1Y 4UH, UK; 4 Department of Genetics, Harvard Medical School, Boston, MA, 02115, USA; 5 Departments of Biology and Biomedical Engineering, Boston University, Boston, MA, 02215, USA

## Abstract

**Background:**

Understanding the response of complex biochemical networks to genetic perturbations and environmental variability is a fundamental challenge in biology. Integration of high-throughput experimental assays and genome-scale computational methods is likely to produce insight otherwise unreachable, but specific examples of such integration have only begun to be explored.

**Results:**

In this study, we measured growth phenotypes of 465 *Saccharomyces cerevisiae *gene deletion mutants under 16 metabolically relevant conditions and integrated them with the corresponding flux balance model predictions. We first used discordance between experimental results and model predictions to guide a stage of experimental refinement, which resulted in a significant improvement in the quality of the experimental data. Next, we used discordance still present in the refined experimental data to assess the reliability of yeast metabolism models under different conditions. In addition to estimating predictive capacity based on growth phenotypes, we sought to explain these discordances by examining predicted flux distributions visualized through a new, freely available platform. This analysis led to insight into the glycerol utilization pathway and the potential effects of metabolic shortcuts on model results. Finally, we used model predictions and experimental data to discriminate between alternative raffinose catabolism routes.

**Conclusions:**

Our study demonstrates how a new level of integration between high throughput measurements and flux balance model predictions can improve understanding of both experimental and computational results. The added value of a joint analysis is a more reliable platform for specific testing of biological hypotheses, such as the catabolic routes of different carbon sources.

## Abstract 2

An iterative approach that integrates high-throughput measurements of yeast deletion mutants and flux balance model predictions improves understanding of both experimental and computational results.

## Background

Recent advances in both high-throughput experimental approaches and computational analysis techniques have provided opportunities to explore biological function at the system level. An area in which this research has flourished is the study of genome-scale metabolic networks. Genome-scale metabolic network stoichiometries, encompassing all known metabolic reactions for a given organism, have been published for a diverse set of organisms, ranging from *Escherichia coli *[1] to human [2]. These network stoichiometries have been used to build quantitative models capable of producing biologically informative and experimentally testable predictions [3,4]. In particular, constraint-based flux balance techniques have established a set of tools for the study of metabolic network behaviors using a steady state approximation and optimality criteria [5,6].

Although flux balance predicted distributions represent rough approximations of the complex reality of cellular metabolism, numerous studies have demonstrated the ability of flux balance models to reproduce various types of experimental results [7,8]. A type of experimental data that has been frequently used for model assessment is the measurement of growth phenotypes under different genetic and environmental backgrounds. The ability to determine growth phenotypes in a high-throughput manner, both experimentally [9,10] and in flux balance models, has contributed to making model comparisons to single deletion mutant growth phenotypes a community standard in the assessment of new models [11-13].

The high predictive capacity of genome-scale models, as inferred from these assessments, has also stimulated their use in studies that address questions currently at the edge of experimental feasibility. These studies have typically taken advantage of the speed of flux balance model computations to make system-level observations that are experimentally challenging or unfeasible. They include the exploration of global patterns of epistasis [14-16], essentiality under combinatorially diverse environmental conditions [17], processes of adaptive or reductive evolution [4,18], complex metabolic engineering optimization [19], and the study of microbial communities [20].

As models become increasingly reliable and useful as discovery tools parallel to experimental methods, new paradigms for the integration of experimental and computational analyses may be explored. It is particularly important to understand how such integration can be used to gain novel biological insight beyond that attainable from independent experimental and modeling studies. Recent integrated analyses have employed iterations of experiments and modeling to drive biological discovery [21-24].

Here, we use model predictions and high-throughput experimental data in a bidirectional and synergistic manner. Specifically, we compare yeast flux balance model predictions with a new compendium of single gene deletion phenotypes under 16 different conditions. Contrary to the usual direction of refinement (whereby models are refined based on experimental data), we start by using computational predictions to identify potential weaknesses in the experimental results. This model-based refinement leads to the identification of several mutant defects, increasing our confidence that discordances are the result of model deficiencies. Based on this refined data, we evaluate the predictive capacity of different modeling frameworks, using an array of statistical metrics, and describe the global features of a yeast metabolism growth phenotype map. In addition, by combining the growth phenotype maps with automated visualization of detailed flux predictions, we present a case study (glycerol utilization) that provides additional insight on the power and limitations of stoichiometric models. Finally, we show how an integrated data analysis approach allows us to discriminate between different hypotheses on the mechanism of raffinose utilization in yeast.

## Results and discussion

Our study combines experimental data and computational predictions of growth phenotypes in the yeast *Saccharomyces cerevisiae*. We experimentally determined growth phenotypes for mutants with single gene deletions of metabolic enzymes under a diverse set of metabolically relevant conditions. Specifically, we focused on 465 of the 892 genes present in one of the stoichiometric models (iFF708; see below, and [25]), which are non-essential for growth in rich glucose medium (YPD) and for which a homozygous diploid deletion mutant was publicly available [10] (Table S1 in Additional data file 2). We used quantitative image analysis of cells replica pinned on agar plates [26] to measure the growth of these strains under 16 environmental conditions that could be mimicked by the models, including different carbon sources, amino acid dropout media, and anaerobic growth (Table 1; Materials and methods). Briefly, the growth of each mutant (assayed using empirically determined parameters of spot size and intensity (Materials and methods and [26]) under each experimental condition is measured relative to its growth under the corresponding control condition. For the purpose of comparison to model predictions, growth rates were discretized into three categories, no growth, slow growth and wild-type growth (Materials and methods). All assays were performed in duplicate and the results agree well between replicates (Materials and methods) and with published results [27] (Figure S2 in Additional data file 1).

**Table 1:** Media conditions implemented in compendium of deletion phenotypes

Condition	Description
SCall	Synthetic complete (SC) medium
SCade	SC, adenine drop out
SCarg	SC, arginine drop out
SCino	SC, inosine drop out
SClys	SC, lysine drop out
SCmet	SC, methionine drop out
SD minimal media	SC, amino acid drop out
YPD	Yeast peptone (YP), glucose is primary carbon source
YPEtOH	YP, ethanol is primary carbon source
YPGal	YP, galactose is primary carbon source
YPGly	YP, glycerol is primary carbon source
YPAC	YP, actetate is primary carbon source
YPLac	YP, lactate is primary carbon source
YPRaff	YP, raffinose is primary carbon source
YPTE no glucose	YP with ergosterol and zymosterol, no glucose
YPTE no O2	YP, with ergosterol and zymosterol, anaerobic condition

Computational analyses of single gene deletion mutants were performed using the steady state approach of flux balance analysis (FBA) and its minimization of metabolic adjustment (MOMA) variant. In these approaches, mass conservation laws translate into linear constraints on reaction rates (fluxes). The additional constraints imposed by gene knockouts are implemented by setting the values of the corresponding fluxes to zero (see Materials and methods). Within the space of flux distributions compatible with such constraints one can identify biologically meaningful states (the flux balance predictions) by computing the optima with respect to an objective function hypothesized to mimic the result of evolutionary or physiological adaptation. FBA predictions of gene deletion effects are often obtained by maximizing biomass production ('growth') [28] whereas in MOMA the fluxes of the knockout are predicted to minimally deviate from their natural wild-type state [29] (see Materials and methods for more details). The search for alternative objective functions constitutes in itself an interesting and active area of research [30-32].

FBA and MOMA calculations were applied to three of the most recent publicly available genome-scale yeast models (iFF708, iLL672 and iND750; Table 2). The iFF708 model was the first genome-scale yeast model, and accounts for 842 reactions in three cellular compartments [25]. The iLL672 model is a modified version of iFF708 that has a more complete biomass definition [13]. The detailed quantification of the molecular components in the biomass reaction is central to model behavior, as it can significantly affect the predicted steady-state flux distribution for any optimization criterion that involves (for example, maximizes) biomass production [11]. Therefore, although the list of reactions in the iFF708 and iLL672 models is largely the same, performance has been shown to vary considerably [13]. The third model is the fully compartmentalized iND750 model, which contains eight cellular compartments and includes an increased number of genes and reactions [11]. Media conditions for all three models were implemented by appropriately setting upper bounds on the fluxes of nutrients into the system (see Table S4 in Additional data file 2 for detailed condition definitions). Importantly, upon setting constraints to implement a particular condition, we verified that the fluxes through the model indicate the proper use of available metabolites (for example, use of intended carbon source).

**Table 2:** Summary of available yeast models

Model	Number of genes	Number of reactions	Number of metabolites	Number of metabolites in biomass reaction that are not included in the biomass in both of the other two models
iFF708	708	842	584	0
iLL672	672	745	636	12
iND750	750	1149	646	2

### Refinement of experimental phenotype data

While most studies only use experimental data to refine models, we started by asking whether the model predictions could be used to improve the quality of experimentally determined phenotypes. Previous comparisons between flux balance predictions and experimental measurements of growth phenotypes for gene deletion strains have reported accuracies upwards of 90% [11,13]. A similar fraction of correct predictions (94%) was obtained in the first comparison of our own experimental data and iFF708 model predictions. These numbers indicate a high predictive capacity of the models, supporting the possibility that computational estimates of phenotypes might serve as a good reference for critically analyzing experimental data. Minimization of experimental errors in this type of study is of great importance for several reasons. First, for the purpose of model refinement based on comparisons with experimental data, experimental inaccuracies could result in either the propagation of model errors or in erroneously fitting models to faulty experiments. Second, such experimental inaccuracies could lead to incorrect conclusions, when used as benchmarks for biological hypothesis testing. Finally, increased accuracy of the experimental data itself is critical in the generation of valid biological insight.

**Figure 1:**
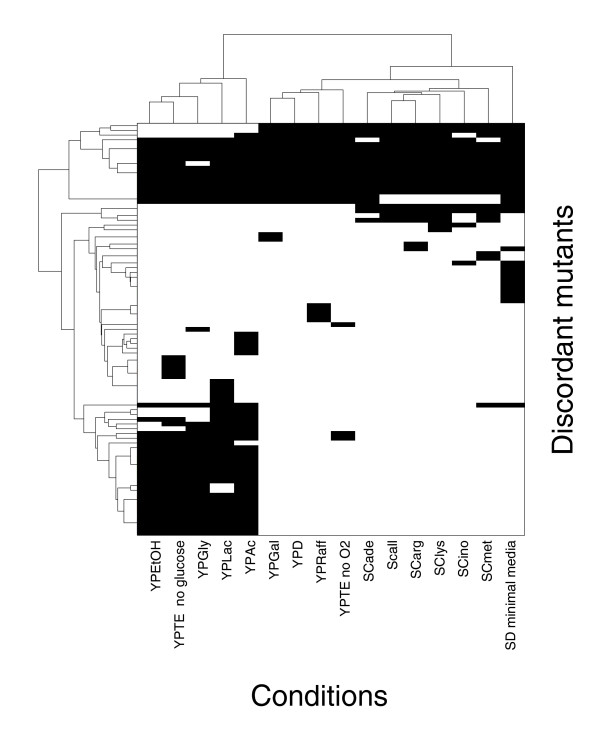
Discordance between experimental phenotypes and iFF708 predictions. Patterns of concordance between experimentally determined phenotypes for 465 single gene deletion mutants and the corresponding predictions made by the iFF708 model were displayed in a clustered binary map for visual inspection (see Materials and methods for details on concordance analysis). Patterns of concordance (white) and discordance (black) are shown for the 87 genes (Table S6 in Additional data file 2) for which the experimental phenotype and the phenotype predicted by either FBA or MOMA disagreed under at least one of the 16 conditions (Table 1). The similarity between genes (vertical axis) and conditions (horizontal axis) is shown as a hierarchical tree view.

We identified 87 mutants for which the experimental phenotype and the computational prediction (with the iFF708 model) disagreed under at least one condition, including 10 mutants that disagreed under all conditions tested (Figure 1). While such a pattern of discordance could be the result of a deficiency in the model, it could be the result of an error in the deletion strain. Preliminary examination of some of the mutants that were discordant across all conditions supported this hypothesis. For example, one of the discordant strains was the deletion mutant for *CDS1*. *CDS1 *encodes the CDP-diacylglycerol synthase and has been previously found to be essential for phospholipid biosynthesis [33]; therefore, it should be essential under all tested conditions. Its essentiality was correctly predicted by the model, but initial experiments showed no growth defects. We extended this model-driven analysis of experimental results to a larger scale, by systematically screening and re-evaluating discordant mutants.

**Figure 2:**
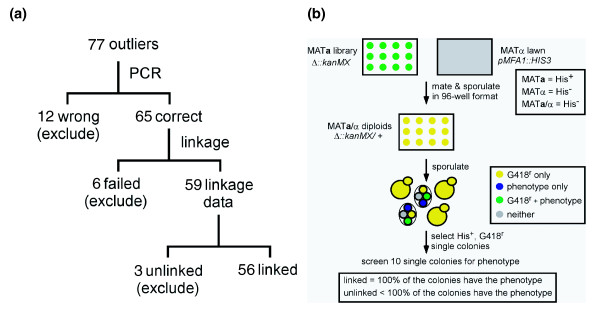
Experimental refinement procedure. **(a) **Overview of procedure and error detection. Beginning with 77 of the 87 deletion mutants whose experimentally measured growth phenotype differed from the model prediction under at least one condition, we tested the presence of the correct deletion mutation by PCR and whether the phenotypes were linked to the gene of interest (Materials and methods). Strains that were incorrect by PCR (12), failed to form haploid progeny in the high throughput linkage method (6), or had phenotypes unlinked to the deletion mutation (3) were excluded from further analysis. **(b) **High-throughput linkage analysis method. MATa haploids containing the gene deletions of interest and gridded in 96-well format are mated to a lawn of the MATα strain containing a HIS3 reporter gene under the control of the MFA1 promoter. This construct only expresses HIS3 in MATa haploid strains, and in this scheme is used to select haploid progeny that have undergone meiosis (half of which will also contain the G418-marked deletion of interest). Following mating and sporulation in 96-well format, tetrads are disrupted by digestion with zymolyase and MATa haploid progeny are selected by plating for single His+, G418r colonies. For each deletion mutant, 10 of these progeny colonies were assayed for the phenotypes of interest. Mutants in which all 10 progeny exhibited the phenotype were considered linked and candidates for further analysis.

Several classes of errors have been observed before in the yeast deletion set, including strain-to-well tracking errors, chromosomal aneuploidy [34], and the presence of phenotypes unlinked to the deletion mutation [35]. To account for these potential issues, we implemented two experimental tests to validate the initial experiments (Figure 2a). First, we used PCR to test whether the strains contained the appropriate mutation (Materials and methods). Of the strains tested, 12 did not contain the correct mutation, and were excluded from further study. We next wanted to verify that the experimental phenotypes observed were linked to the deletion mutation, and not the result of secondary mutations. To facilitate genetic linkage analysis with a large number of strains, we developed a high throughput linkage strategy (Materials and methods). Briefly, this method (Figure 2b) uses a *HIS3 *reporter gene placed under the transcriptional control of the *MFA1 *promoter to allow selection for the haploid (MATa) products of meiosis following mating and sporulation [36]. The availability of this selection replaces the labor-intensive step of conventional linkage analysis, i.e. tetrad dissection, with a simple colony selection procedure. Mutations were considered linked to the phenotype of interest if 100% of the 10 haploid colonies screened displayed that phenotype. Strains that were resistant to analysis due to defects in mating, sporulation, or auxotrophies that interfere with the selection of single colonies were excluded from further analysis. Of the 69 deletion strains screened in this manner, 66 showed phenotypes that were genetically linked to the drug resistance marked deletion mutation; the remainder were removed from further analysis. In the cases where linked phenotypes of the haploid progeny did not agree with the phenotype of the original diploid, the haploid phenotype was used for comparison with the model prediction.

**Figure 3:**
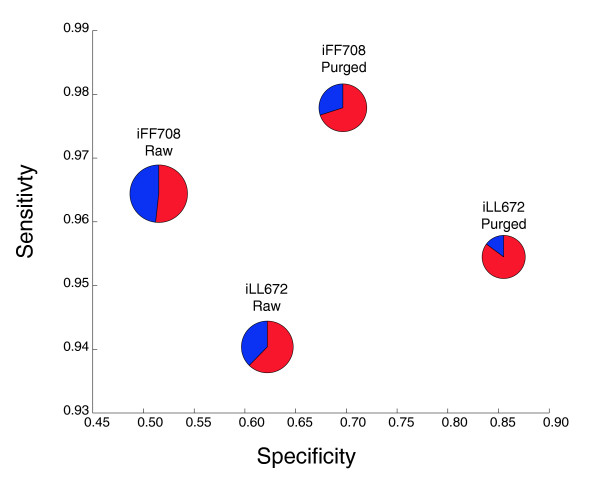
Sensitivities and specificities of iFF708 and iLL672 models before and after the model-directed experimental refinement process. To assess the impact of the experimental refinement process, we plot here the sensitivities (ordinate) and specificities (abscissa) of predictions made by the iFF708 and iLL672 models, before and after refinement. Each combination of a model (iFF708 or iLL672) and an experimental data set (before or after refinement) is represented by a pie chart, with the two slices representing the number of essential genes correctly (red) or incorrectly (blue) predicted by the model. The size of the pies represents the relative numbers of experimental essential phenotypes that are present among a given model's gene set. It can be seen that, for both models, the sensitivities and specificities are greater with the refined data set. Note that while experimental refinement was directed by discordance with the iFF708 model's predictions, the increase in concordance is also significant for the iLL672 model's predictions.

While our experimental validation of discordant mutants identified several faulty strains, it is likely that additional experimental error still went undetected. Yet, this refinement process did have a significant impact on the quality of the data set. In order to quantify the impact of the experimental refinement, we compared the concordance of the original (Table S1 in Additional data file 2) and refined (Table S3 in Additional data file 2) phenotypic measurements with model predictions. Figure 3 shows this comparison for the iFF708 model, the model used to select mutants tested for errors, and for the iLL672 model, a modified version of the yeast model [13]. It is clear that both models show improvement in both sensitivity and specificity after the refinement, indicating an increase in concordance. Notably, although the iFF708 model was used to originally define discordance, and therefore dictated which strains were tested for errors, the iLL672 model showed similar improvement in both sensitivity and specificity. This supports the assertion that the improved concordance is due to the identification of the correct phenotypes and not just a consequence of retesting only discordant mutants resulting in fitting experimental phenotypes with model predictions. The non-random nature of model directed identification of experimental errors was further implied by the observation that although only approximately 20% of mutants were retested, there was a reduction in the number of false positive predictions by the iLL672 model of greater than 70%.

### A clustered map of mutant phenotypes reveals diversity of metabolic behaviors

**Figure 4:**
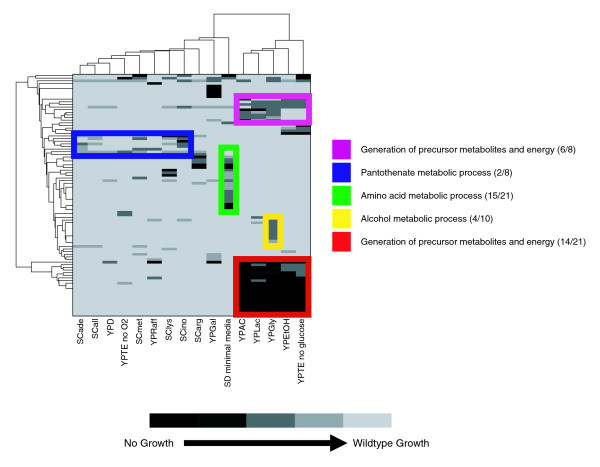
Two-dimensional hierarchical clustering of refined experimental phenotypes. Experimental phenotypic profiles for those strains that showed reduced growth under at least one condition were clustered using two-dimensional hierarchical clustering. The rows are different genes and the columns are the 16 experimental conditions present in the current data set. Each entry is representative of the phenotype of the knockout of a particular gene under a particular condition, with more severe phenotypes being represented with darker shades of gray. Prominent clusters have been boxed, and the most significantly enriched Gene Ontology biological process terms among the genes in each cluster are noted to the right. This representation allowed for several immediate observations. For example, it can be seen that the red and purple clusters primarily contain mutants that show a phenotype only under non-fermentable carbon sources. Fitting expectations, process analysis revealed that the majority of the genes in these clusters participate in respiratory function. Another observation that fits with biological intuition is the enrichment of amino acid biosynthetic genes in the green cluster, which encompasses only the minimal media condition. Given that the other conditions lack, at most, only an individual amino acid, it fits with expectations that most amino acid biosynthetic genes should be essential only in the condition where all amino acids, except those for which the utilized strain cannot produce (for example, histidine and leucine), are absent. The remaining clusters capture more diverse sets of genes, and individual Gene Ontology terms are not as illuminating as to the metabolic challenges faced under the conditions encompassed by the given clusters.

Using our refined set of experimental growth phenotypes, we next examined the patterns of essentiality under different conditions. Given the premise that each genetic unit should provide some fitness benefit under some habitually encountered condition [37,38], the percentage of genes found to be essential for wild-type growth under the tested conditions should inform us as to the breadth of metabolic challenges captured by our experiment. Following discretization of the growth rates into categories of no growth, slow growth, and normal growth (see Materials and methods), a quick overview of the data revealed that 92 of the 444 deletion mutants tested displayed sub-wild-type growth under at least one of the conditions tested. This suggests that we have sampled a significant slice of the evolutionarily relevant metabolic condition space for *S. cerevisiae*. Next, two-dimensional hierarchical clustering was performed in order to group together conditions that require similar sets of enzymes. The clustered heatmap representation of the discretized data shown in Figure 4 provides new insight, hard to gain from the unclustered map. First, it is evident that the growth defects in the five non-fermentable carbon source conditions (YPEtOH, YPAc, YPGly, YPLac, and YPTE) are very similar. As expected, the common genes relate to cellular respiration, participating in processes such as electron transport, oxidative phosphorylation and biosynthesis of electron transport associated cofactors. A second striking observation is that although there are many genes whose deletion resulted in severe phenotypic effects in glucose minimal media, very few of them resulted in the complete abolition of growth. More detailed analysis revealed that most of these genes are involved in amino acid biosynthesis (Figure 4, green box). One may speculate that the ability of yeast strains with defects in amino acid biosynthesis to grow without supplementation of amino acids suggests an overall robustness in these pathways.

### Flux balance models predict essentiality under diverse conditions

After utilizing the model results to refine the experimental phenotypes, we next took advantage of this refined data set to build a benchmark for assessing model performance across different conditions through multiple statistical metrics. In addition to assessing model predictions using the current compendium of deletion mutant data, we also included mutants that have no growth under YPD, so that we could gain a more complete picture of model performance. Specifically, genes required for growth under YPD were assumed to be required under all conditions. While this assumption is not universally valid, it is likely to be largely correct due to the fact that most nutrients provided under other conditions are also provided under YPD.

**Figure 5:**
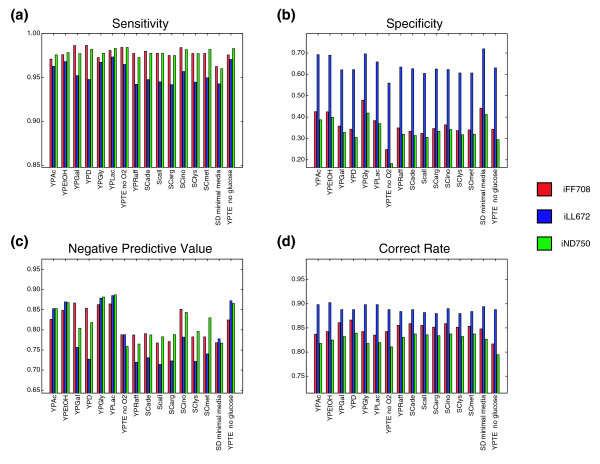
Overall model performances, including YPD essential genes. Predictive performance of the iFF708 (red), iLL672 (blue) and iND750 (green) models are shown for the 16 different conditions present in the current data set. For the calculations of the different metrics, true positive (TP) predictions were regarded as experimentally viable genes predicted to be viable, false positives (FP) as experimentally essential genes predicted to be viable, true negatives (TN) as experimentally essential genes predicted to be essential, and false negatives (FN) as experimentally viable genes predicted to be essential. Calculations of **(a) **sensitivity (TP/(TP + FN)), **(b) **specificity (TN/(TN + FP)), **(c) **negative predictive value (TN/(TN + FN)) and **(d) **correct rate ((TP + TN)/(TP + TN + FP + FN)) were done with genes essential under YPD considered to be essential under all conditions. Assessing models using a variety of metrics reveals that the models differ in their abilities to identify viable and unviable mutants. For example, the higher specificity of the iLL672 model under all conditions indicates that it identifies the largest proportion of essential genes. On the other hand, the higher negative predictive value of the iFF708 and iND750 models demonstrates that the percentage of correct essential predictions is lowest using the iLL672 model. This trade-off suggests that different models may be preferable for use in different applications, depending on the relative impact of false positives and false negatives.

A common metric for quantifying the ability of metabolic network models to predict the consequences of single gene deletions is the overall fraction of correctly predicted growth phenotypes, i.e. the number of correct predictions divided by the total number of predictions. A previously reported issue with this metric [13,39] is that there is an inherent imbalance in essential phenotypes. Specifically, viable deletion mutants are roughly four times more abundant than inviable ones. The result of this bias is that overall prediction accuracy does a poor job of communicating the true nature of the model predictions, as essential mutants are more difficult to identify than viable ones. This effect can be seen in Figure 5, where the three different yeast models are compared based on their correct rates (Figure 5d), and a variety of other metrics. The iLL672 model is better than the other models by as little as 2% under some conditions when considering correct rate, but when judged by the percent of essential genes identified (specificity; Figure 5b), the iLL672 model is better by no less than 22% under any condition. Therefore, if one values the ability to predict a maximal number of essential genes, then specificity is the most informative metric, as it clearly separates the models. On the other hand, for other applications of metabolic models, it is not the number of essential genes identified that is most important, but the reliability of those essentiality predictions. For example, if a model is being used to identify putative drug targets, then minimizing experimental exploration of candidate targets to a highly accurate set of essential predictions would be ideal. In that case one would be concerned with the negative predictive value, which represents the accuracy of essential predictions (see Figure 5 legend for definitions of metrics). In the case of the three yeast models, the determination of which model is best is completely reversed when considering negative predictive value (Figure 5c), as the iND750 model has the highest negative predictive value under the majority of conditions. The different conclusions reached depending on the metric used suggest that a single metric is not sufficient to compare the models, but that an appropriate metric should be relied upon depending on the particular application of the model.

The tendency for the different models to vary in their relative performance when considering different metrics can largely be explained by considering their previously mentioned differences. For example, the observation that the iLL672 model predicts more essential genes than the other models is predominantly due to its altered biomass definition. Specifically, the fact that the biomass definition for the iLL672 model contains 12 additional metabolites dictates that genes in pathways leading to the production of those metabolites will be required for growth. Therefore, in the absence of an exogenous supply of a given biomass metabolite, the corresponding biosynthetic genes will be predicted as being essential. It should be noted that because the definition of biomass for a given model is independent of the condition, changes in the biomass definition will not improve the ability of the model to differentiate between the metabolic requirements under different conditions. For instance, ubiquinol, a cofactor required for respiratory function, is one of the 12 metabolites added to the biomass definition for the iLL672 model. As a consequence of this imposed requirement for ubiquinol, ubiquinol biosynthetic genes are correctly predicted to be essential in the presence of non-fermentable carbon sources, where respiratory function is required. On the other hand, in the presence of fermentable carbon sources these genes are incorrectly called essential, as respiratory function is no longer essential to growth (Figure S1 in Additional data file 1).

### Model predictions of condition-specific essential genes

**Figure 6:**
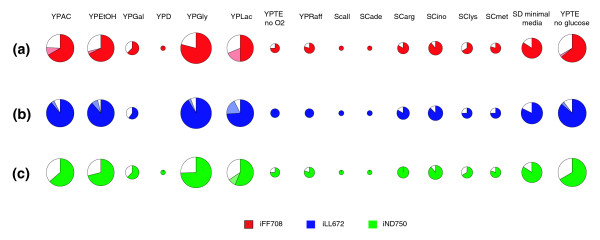
Condition-specific essential gene identification by the three yeast models. The models are assessed here solely on their ability to identify genes that are essential under a given condition and not essential under YPD. The size of the pies is proportional to the number of genes essential under a given condition relative to other conditions. The largest number of condition-specific essential genes was the 43 found under YPAC, and hence the essential genes for this condition are represented by the largest pies. The number of essential genes identified under each condition with FBA is shown for the iFF708 (red), iLL672 (blue) and iND750 (green) models. Additional essential genes identified using MOMA are shown in a lighter shade and essential genes not identified are represented by the white slices. In all models, under virtually all conditions, the majority of condition specific essential genes are identified, indicating that the predictive abilities of the models are robust to different media conditions.

A more focused approach for assessing the ability of the models to accurately capture diverse cellular behaviors is to consider only the propensity of the models to identify condition-specific essential genes. Therefore, for the current analysis we did not include genes required for growth under YPD. Figure 6 shows the proportion of condition specific essential genes identified by the models under each of the conditions tested. Overall, when mutant viability was determined using the assumption of maximum growth, between 70% and 85% of the condition-specific essential genes were identified by the three models. Importantly, the high mean percentage of condition-specific essential genes identified was achieved by consistent performance under most conditions, as opposed to disproportionately high percentages in a few conditions.

In addition to tabulating the number of condition-specific essential genes identified using the assumption of maximal mutant growth, we determined how many additional genes could be identified by implementing the alternative optimization criterion of MOMA [29]. Rather than assuming that the flux distribution of a deletion mutant will necessarily be optimal for growth, MOMA is based on the hypothesis that the mutant flux distribution will be minimally distant from that of the wild type. This approach is motivated by the fact that one should not necessarily expect an organism to respond optimally to a gene deletion. Rather, in the absence of an evolved response to the sudden removal of a gene, one might hypothesize that the metabolic network will tend to stay close to the unperturbed steady state. The MOMA hypothesis has been supported by experimental studies in yeast, as well as other organisms, where the flux response to gene deletions was determined using C13 tracer experiments [40,41]. These studies observed a local rerouting of metabolic fluxes around the reactions compromised by gene deletions in viable deletion mutants, consistent with the MOMA hypothesis of minimal flux redistribution. An important step in the implementation of MOMA is the selection of wild-type flux predictions, from which the distance is minimized. Ideally, one should use a wild-type solution constrained by experimental flux measurements [13,29], but experimental flux measurements were not available for all the studied conditions. Therefore, we used the FBA predicted optimal solution, with a secondary optimization that minimizes the sum of the absolute values of the fluxes. This secondary optimization is necessary to select a specific set of fluxes among the alternative flux solutions equally optimal for growth. The biological relevance of this flux minimization criterion has been previously reported [42,43].

Focusing on our condition specific essentiality predictions, we found that utilization of MOMA led to the correct identification of an additional six (average among three models) condition-specific essential genes, beyond the set identified using the assumption of optimality (Figure 6). Using a slightly more stringent definition for model agreement with experimental results (see Materials and methods), we found that, on average, 14 condition-specific essential genes are identified using MOMA with the different models, relative to FBA. Especially striking was the observation that under the condition when glycerol is provided as the primary carbon source, 9 and 10 additional essential genes were identified by MOMA in the iFF708 and iND750 models, respectively. Additional inspection revealed that these additional essential genes identified by MOMA under glycerol conditions all functioned in respiratory metabolism.

### Automated visualization for detailed assessment of flux predictions

Assessment of the concordance between computationally and experimentally determined mutant growth phenotypes provides a coarse evaluation of a model's propensity to correctly reproduce metabolic function. To more rigorously establish that a metabolic model accurately depicts metabolic behavior under a particular condition, one must judge the accuracy of the predicted flux distribution underlying the predicted growth rate [29,30]. Unfortunately, experimentally measured fluxes are only available for a few organisms, under a small number of conditions, making global model assessment in this manner incomplete. One can, however, employ a more qualitative assessment by simply verifying that the predicted fluxes match biological knowledge, as supported by other types of data. A major hurdle in making such a qualitative assessment is the difficulty of automatically visualizing metabolic fluxes in a way that would allow immediate biological insights. While static networks, as well as platform-specific or model-specific visualization methods, are widely available [44-48], a general platform for metabolic network visualization is still lacking. To address this problem, we developed a visualization pipeline, which holds the potential to evolve into a general purpose platform. Our metabolic flux representation pipeline uses the freely downloadable VisANT network visualization software [49]. Specifically, we used VisANT to create a standard layout of the reactions of central energy metabolism that are present in the iFF708 and iLL672 models, and then loaded previously computed flux distributions for visual analysis (see Materials and methods for details on network visualization). As a supplement to this work we have provided an online tool that allows for interactive visualization of flux distributions predicted by the iLL672 model for all single deletion mutants [50].

### Detailed evaluation of fluxes under glycerol growth condition gives insight into model behavior

We used our visualization framework to explore the underlying basis of some of the model predictions. Specifically, we examined in detail the fluxes predicted by the iFF708 model for mutants in the respiratory chain under glycerol conditions. As described previously, these mutants were incorrectly predicted to be able to grow under this condition using the FBA assumption of optimality, and correctly predicted as non-growers using MOMA. Examination of these mutants was especially interesting as it had the potential to provide insight into why yeast does not utilize the predicted optimal metabolic route when confronted with such gene deletions. Previous studies have indeed found that *E. coli *grows suboptimally in glycerol, and that the FBA-predicted optimum is achieved only upon several generations of *in vitro *evolution [4]. The mutations underlying the improved glycerol growth phenotype caused major regulatory changes, likely detrimental to growth under more commonly encountered conditions, and therefore absent in the wild type [51,52].

**Figure 7:**
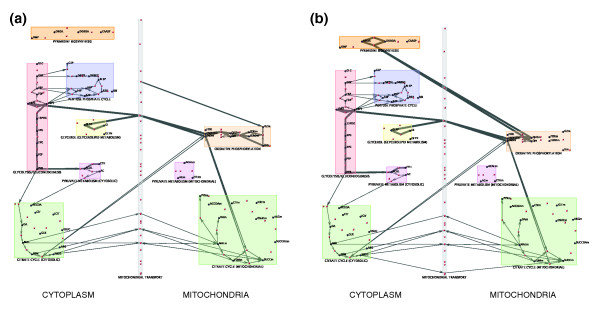
Fluxes through central energy metabolism in the iFF708 model under glycerol conditions. The VisANT network visualization software (see Materials and methods) was utilized to display flux distributions predicted by the iFF708 model as edges on a graph containing the reactions and metabolites participating in yeast central energy metabolism. For both networks, red nodes represent reactions and blue nodes represent metabolites. Edges between reactions and metabolites are indicative of metabolites being either reactants or products in the given reaction. The thickness of a particular edge is indicative of the relative flux through the reaction, where all fluxes are normalized by the maximal flux through an individual network. Predicted flux distributions are shown for **(a) **the wild type under glycerol conditions and **(b) **a mutant lacking complex III of the electron transport chain under glycerol conditions. Mutant flux distributions were computed here using the FBA assumption of optimal growth. The most prominent difference between the mutant and wild-type fluxes is the diversion of flux from the respiratory chain to the reaction in pyrimidine biosynthesis catalyzed by Ura1. This incorrect rerouting of flux in the respiratory mutant results in the incorrect prediction by the model that respiratory function is not essential when glycerol is the primary carbon source.

The flux distribution predicted by the iFF708 model in glycerol with respiratory function intact (Figure 7a) demonstrates that the route for glycerol catabolism utilized in the model simulation matches the canonical pathway described in biological pathway databases [53]. Briefly, glycerol is first phosphorylated by glycerol kinase and the resulting glycerol-3-phosphate is converted to dihydroxyacetone phosphate. This second step is associated with the donation of electrons from glycerol-3-phosphate to the electron transport chain (ETC) via flavin adenine dinucleotide (FAD). Next, the dihydroxyacetone phosphate enters glycolysis and gluconeogenesis to meet the cells biosynthetic needs. A respiratory deficient mutant should be unable to grow with glycerol as the sole carbon source, because there is no means by which FAD can be re-oxidized, and without FAD available as an electron acceptor, glycerol catabolism cannot proceed.

To elucidate the route by which FBA circumvents the apparent redox imbalance that should occur in the absence of respiratory function, we visualized the flux distribution predicted by FBA when complex III of the ETC was knocked out. As can be seen in Figure 7b, the flux entering the ETC has been diverted from complex III to another reaction, which is catalyzed by Ura1. Ura1 catalyzes a redox reaction that is the fourth step in pyrimidine biosynthesis [54]. In the iFF708 model this reaction utilizes the ETC intermediate ubiquinone as an electron acceptor or donor depending on the direction in which the reaction proceeds. While it is common in other yeast species for the reaction catalyzed by the ortholog of Ura1 to utilize the ETC as an electron donor/acceptor, in *S. cerevisiae *the Ura1 enzyme is cytosolic, and uses fumurate as an electron acceptor [54,55]. Therefore, we conclude that the redox imbalance is averted in the FBA solution through the utilization of a reaction that is misrepresented in the model. This conclusion was further confirmed by the observation that when the Ura1 reaction is excluded from the model, FBA correctly predicted the inability of respiratory mutants to grow under glycerol conditions. This was a critical validation, as it excluded the possibility that there were alternative optimal flux solutions that did not use the Ura1 reaction [56].

The finding that a subset of the predictions discordant between FBA and MOMA were in this case due to an inaccurate model reaction, and not a biologically meaningful difference between MOMA and FBA, illustrates the value of verifying model-based conclusions at the level of fluxes. Analysis of predicted fluxes revealed that the discordant predictions were attributable to the propensity of FBA, and not MOMA, to drastically reroute fluxes so as to utilize the incorrect model reaction. While the growth maximization objective exploits the misrepresented flux, the minimal adjustment objective, lacking the foresight of how to rearrange fluxes optimally, identifies a solution that uses this flux minimally, and fails to produce some biomass components. Had we simply taken the differing phenotypic predictions between FBA and MOMA at face value, we might have reached the conclusion that their predictions differed due to the fact that *S. cerevisiae *does not exhibit optimal responses to gene deletions in the respiratory chain under glycerol conditions, possibly because glycerol is a non-preferred carbon source. This case illustrates the fact that a correct interpretation of model results may require a detailed examination of the flux predictions underlying the phenotypic predictions. While it is unreasonable to examine all flux predictions in such detail, our results suggest that there is value in validating major conclusions at this level.

### Discriminating between alternative pathways for raffinose utilization

**Figure 8:**
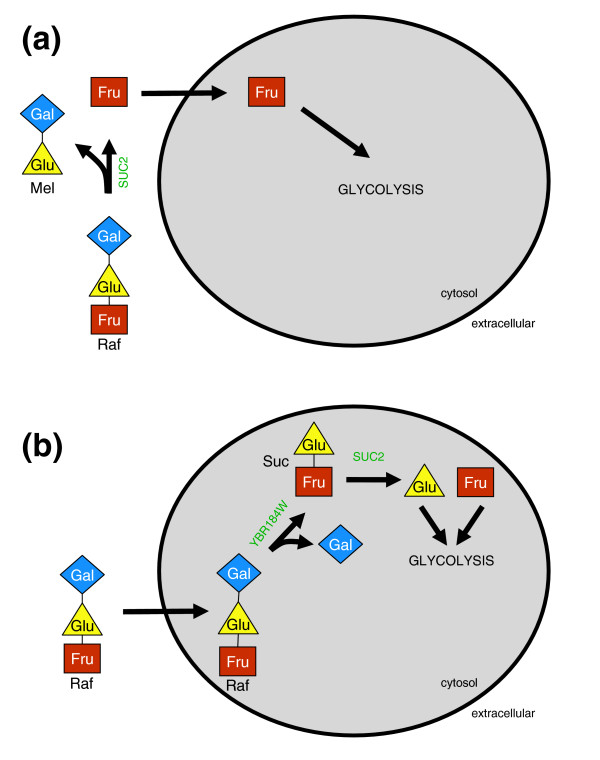
Proposed routes for raffinose utilization in *S. cerevisiae*. **(a) **Based on annotation in KEGG and reactions already present in the yeast models, our initial implementation of the raffinose utilization pathway began with the intracellular cleavage of the *α*-galactosidic bond in raffinose (blue diamond + yellow triangle + brown square) by the protein product of YBR184W to produce sucrose (yellow triangle + brown square) and galactose (blue diamond). Subsequently, the *β*-fructosidic bond in sucrose is cleaved by SUC2, resulting in the formation of glucose (yellow triangle) and fructose (brown square). Finally, the glucose and fructose produced from sucrose hydrolysis can enter glycolysis and meet cells' metabolic needs. **(b) **Based on literature citing the lack of an *α*-galactosidase in the strain of *S. cerevisiae *studied here, we implemented a raffinose utilization pathway lacking the *α*-galactosidase reaction. The two different pathways for raffinose utilization were each implemented separately in the iLL672 model, and assessed based on the relative concordance of the phenotypic predictions made by the different builds, with the experimentally determined gene deletion phenotypes. We found an increased concordance with the iLL672 build lacking YBR184W, suggesting that the pathway depicted in (b) is correct.

In addition to their utility in identifying potential errors, patterns of concordance and discordance between experimental data and models can be exploited as a means of generating biological insight. For example, an approach for elucidating the structure underlying biological systems is to build a set of models differing in their representation of the system of interest, and identify the model that can best reproduce experimental data. While this approach has been applied with other modeling platforms and data [23,57], to our knowledge it has not been exploited with genome scale models of metabolism and complementary genome-scale phenotype data. As a test of this approach, we set out to assess two alternative hypotheses for raffinose utilization in yeast, which have been reported in pathway databases and the literature. Raffinose is a tri-saccharide composed of galactose, glucose and fructose, and is the second most abundant carbohydrate found in nature, after sucrose [58]. The first pathway we evaluated for raffinose utilization is illustrated in Figure 8a, and is based on the relevant KEGG pathway [59] along with reactions present in the yeast models. This pathway includes two key reactions catalyzed by the protein products of *YBR184W *and *YIL162W *(*SUC2*). The reactions dependant on Ybr184w and Suc2 cleave the *α*-galactosidic and *β*-fructosidic bonds in raffinose, respectively, resulting in the release of all three saccharide units. The second pathway evaluated is illustrated in Figure 8b, and is based on several literature sources [58,60,61]. The critical difference from the pathway in Figure 8a is the absence of the *α*-galactosidase, Ybr184w. In the absence of the *α*-galactosidic bond cleavage reaction, raffinose is converted to fructose and melibiose. While *S. cerevisiae *readily metabolizes fructose, the strain of *S. cerevisiae *used in this study is unable to metabolize melibiose [62], an observation that has been supported by experimental carbon source removal assays [61]. The finding that yeast is unable to utilize melibiose provides strong evidence that the second pathway is indeed correct and that the sources reporting the first pathway are incorrect. We used this as an opportunity to explore the capacity of model-experiment comparisons to serve as an avenue for biological hypothesis testing.

As the central difference between the two hypothetical pathways is the presence of an *α*-galactosidase enzyme, our analysis focused on determining whether available experimental data and models support the presence of this enzymatic activity. In order to clearly determine the contribution of model predictions to this analysis, we started by consulting genome-scale experimental data alone, independent of the models. We hence first asked whether the phenotypic data support the participation of the putative *α*-galactosidase, Ybr184w, in raffinose utilization. To this end we identified those genes that were experimentally determined to be essential only under the raffinose condition. This analysis revealed that the only gene uniquely essential under this condition is the *β*-fructosidase, Suc2. The fact that only *SUC2 *was identified as essential, and not *YBR184W*, is a first indication that Ybr184w does not participate in raffinose utilization in yeast.

To gain further insight into the role of *SUC2 *and *YBR184W *in raffinose metabolism, we looked at the expression of these two genes in the presence of a variety of carbon sources, utilizing a publicly available set of mRNA micorarray experiments [63]. Examination of the expression pattern of SUC2 mRNA revealed that it has almost a two-fold increase in expression in the presence of raffinose, relative to the other carbon sources tested. In contrast, YBR184W mRNA does not show an increase in expression in the presence of raffinose, or any of the other carbon sources tested. The lack of an altered expression level or a growth defect for the *ybr184w *deletion mutant in the presence of raffinose suggests that *YBR184W *does not participate in raffinose utilization and is likely misannotated as an *α*-galactosidase.

While the analyses performed with phenotype and expression data strongly suggest that *YBR184W *does not function in raffinose utilization, these analyses do not preclude the presence of an as yet unannotated *α*-galactosidase. While additional hypothesis-driven experiments could be performed to test for *α*-galactosidase activity, we next asked whether utilization of the model predictions, in conjunction with the phenotype data, could eliminate the need for additional experimentation. In order to assess whether or not yeast utilizes an *α*-galactosidase during raffinose metabolism, we used the iLL672 model as a framework in which alternative hypotheses for the metabolic fate of raffinose could be tested. To determine which route of raffinose metabolism is more consistent with experimental observation, we compared the concordance of experimentally determined single gene deletion phenotypes in the presence of raffinose, with the predictions made by the two versions of the iLL672 model, differing only by the presence or absence of an *α*-galactosidase reaction. We found that the iLL672 build lacking an *α*-galactosidase correctly predicted the phenotypes for six genes that were predicted incorrectly by the model in which an *α*-galactosidase was present. The six genes whose deletion phenotype were correctly predicted by the model lacking the *α*-galactosidase included four genes correctly predicted as not required for growth in raffinose (*YBR184W*, *GAL1*, *GAL7*, *GAL10*) and two genes correctly predicted as required for growth in raffinose (*SUC2*, *PGI1*). Descriptions of these genes and the source of the differential prediction between the two model builds are detailed in Table 3.

**Table 3:** Deletion phenotypes predicted differently under raffinose conditions by iLL672 builds with and without *α*-galactosidase activity

Gene	Experimental phenotype of deletion under YPRaff	Predicted phenotype of deletion mutant made by iLL672 build with *α*-galctosidase	Predicted phenotype of deletion mutant made by iLL672 build without *α*-galctosidase	Reason for incorrect prediction made by iLL672 build with *α*-galactosidase activity
*Gal1*	Viable	Unviable	Viable	Galactose is produced when *α*-galactosidic bond in raffinose is cleaved. Therefore, in build with *α*-galactosidase, Gal genes are required to metabolize galactose produced during raffinose catabolism
*Gal7*	Viable	Unviable	Viable	See explanation for Gal1
*Gal10*	Viable	Unviable	Viable	See explanation for Gal1
*YBR184W*	Viable	Unviable	Viable	YBR184W is the putative *α*-galactosidase and is essential by definition of the model build
*Suc2*	Unviable	Viable	Unviable	The galactose produced when the *α*-galactosidic bond is cleaved can be used as the primary carbon source, with the remaining sucrose unit being excreted
*Pgi1*	Unviable	Viable	Unviable	Pgi1 catalyzes the reversible conversion of glucose-6-phosphate to fructose-6-phosphate in glycolysis and gluconeogenesis. The pentose phosphate pathway can be used to bypass the deletion of this gene to maintain glycolytic function Without the *α*-galactosidase only the fructose unit of raffinose is usable, making Pgi1 essential for its role in gluconeogenesis

Evaluation of the two model builds based on their relative concordance with the phenotype data provided strong support for the build lacking an *α*-galactosidase, in agreement with previous reports [61,62]. While in this instance standard biochemical assays could have been performed to differentiate between the two proposed routes for raffinose catabolism, this may not be true or easy in general. Therefore, genome-scale metabolic models and data can be thought of as broadly applicable tools, usable for hypothesis testing in conjunction with molecular biology tools.

While the cases examined above illustrate specific examples of how the combination of experimental and computational data can help gain biological insight, we expect that most discrepancies between the refined experimental data and model predictions could yield additional useful information and novel testable hypotheses. Guidelines on how to interpret the results of the comparison between experimental phenotype data and FBA and MOMA model predictions, along with potential strategies for validation, are presented in Table S7 in Additional data file 2. By applying these guidelines systematically to our data, we generated an easily accessible list of all inconsistencies unresolved between model and experiment, together with possible biological interpretations (Table S8 in Additional data file 2). Paired with the online interactive flux visualization tool described above, this list might be used 'on demand' to help understand specific pathways, or as the starting point for further systematic analyses, such as integration with other data sets.

## Conclusions

By generating a yeast compendium of experimentally determined phenotypes for single gene deletion mutants of metabolic genes and predictions from stoichiometric models, we explored ways in which genome scale experimentation and modeling can be utilized synergistically. We found that utilizing the models to explore the experimental data proved useful in both experimental quality control and hypothesis testing. These analyses demonstrated that genome-scale models are not only useful in addressing questions beyond experimental tractability, but can function in parallel with experimentation to drive biological discovery. At the same time, our analysis of predicted intracellular fluxes was essential for drawing accurate conclusions in cases otherwise wrongly predicted based on phenotype comparisons, indicating that all outcomes of flux balance models should be critically analyzed for biological interpretation.

Genome-scale studies often aim to systematically extract biological information from model-data comparisons. As such systematic comparisons are increasingly common in different organisms, it is important to understand how experimental data imperfection, stoichiometric model choice and output interpretation can affect biological conclusions. We hope that our assessment of models and data, based on a new publicly available data set will provide a platform for more informed future systematic understanding. Moreover, increased awareness of the advantages and potential drawbacks of these approaches should help promote the fact that large-scale systems biology methods are not limited to providing high-level insights, but can also be used to expand our knowledge of specific biological pathways.

## Materials and methods

### Strains and media

We measured the growth phenotypes of the 465 homozygous diploid mutants (Table S1 in Additional data file 1) present in both the yeast FBA model [25] and the yeast deletion set [10]. Each mutant contains a precise deletion of an open reading frame in the strain BY4743 (MAT**a**/*α his3Δ1*/*his3Δ1*, *leu2Δ0*/*leu2Δ0*, *ura3Δ0*/*ura3Δ0*, *met15Δ0*/*MET15*, *lys2Δ0*/*LYS2*) [10]. The growth of each homozygous deletion mutant was examined at 30°C under 16 conditions. Unless indicated, all media recipes are referenced in [64]. Six rich (YP) media conditions were used to test carbon source utilization, including YPGly (3% glycerol), YPLac (2% lactate), YPEtOH (2% ethanol), YPOAc (2% potassium actetate), YPGal (2% galactose/1 mg/ml antimycin A), YPRaff (2% raffinose/1 mg/ml antimycin A). Anaerobic growth was measured using YPDTE (2% glucose/20 mg/ml ergosterol/0.5% Tween 80/0.5% ethanol) media [65] and the BBL GasPak Plus anaerobic system catalyst and indicator strips (Becton/Dickinson, Franklin Lakes, NJ, USA). We also tested YPTE (20 mg/ml ergosterol/0.5% Tween 80/0.5% ethanol) under aerobic conditions as an example of a poorly characterized condition that could be metabolically modeled. Six minimal media were used to measure various auxotrophies, including lysine (SC-Lys), adenine (SC-Ade), tryptophan (SC-Trp), inositol (SC-Ino), arginine (SC-Arg), and minimal medium supplemented with only the nutrients required by the parental strain (SD + histidine, leucine, and uracil) [10]. YPD and SC complete media [10] were used as controls for the growth rate of each mutant under rich and minimal media conditions, respectively.

### Growth assays

The 465 strains of interest were selected from the 4,710 strain homozygous diploid yeast deletion set (Open Biosystems, Huntsville, AL, USA) and re-arrayed into a 96-well format. Strains in 96-well format were grown to saturation in liquid YPD, transferred to agar plates containing each of the 16 media conditions by replica pinning, and grown at 30°C until the wild-type controls had reached a sufficient growth level. All conditions were performed in duplicate. Mutant growth under each condition was quantified using an automated system that uses image analysis software to quantify strain growth [26]. Plates were digitally photographed using a GelDoc Station (Bio-Rad Laboratories, Hercules, CA, USA) with images saved as 8-bit TIFF images and converted to 16-bit TIFFs using Adobe Photoshop. Images were batch processed using the GenePix image analysis software (Molecular Devices, Sunnyvale, CA, USA), and data corresponding to the 96 spots per plate were saved as tab-delimited text files. General growth differences between plates or conditions were normalized by assuming that only a small subset of spots would deviate from wild-type growth and calculating the average diameter and intensity measurements of all spots on a plate. Spots differing from this average by empirically determined standard deviations (SD) were deemed slow-growers or non-growers. Spots scored as absent by the GenePix scoring algorithm or with a diameter of 40 pixels or less were scored as non-growers, spots with intensities less than 2.5 SD and diameters less than 1 SD or with intensities less than 1 SD and diameters less than 3 SD were scored as slow growers, all other values received a wild-type growth score. The relative growth of each strain under an experimental condition was then normalized by comparison to its growth on the control medium (YPD or SC), distinguishing condition-specific growth defects from general slow growth. For the purpose of comparison to the model predictions, growth rates were discretized into wild-type growth (2) slow growth (1), and no growth (0).

### Error estimates

The variability and sensitivity of this method for determining mutant growth phenotypes have been evaluated in a prior study [26]. To evaluate the variation and potential error in this specific data set, we performed two sets of analyses. First, we analyzed the variability between replicates. After filtering systematic errors due to the failure of the strain to grow on the YPD or SD control plate, less than 3% of the data points in the initial data set differed between replicates. Second, we compared our growth values to published results [27] for the same strains under the same conditions, but measured using a different experimental system, competitive growth assayed by microarray hybridization (Figure S2 in Additional data file 1).

### Selection of mutants for experimental refinement

Mutants were selected for experimental refinement on the basis of disagreement of the experimental growth phenotype with the corresponding prediction made by the iFF708 model, under at least one of the 16 conditions. A prediction was deemed discordant if the difference between mean growth rate of the two duplicate growth measurements differed from either the MOMA or FBA predicted growth rate by more than a selected threshold (see below). A total of 87 mutants were flagged with this approach, of which 77 were retested with PCR and linkage analysis (see next section).

### Confirming phenotypes by PCR and linkage

To confirm the presence of the correct gene deletion in the original strain screened, the homozygous diploid mutant was examined by PCR (Table S2 in Additional data file 1). To test whether the observed phenotypes were linked to the deleted gene, we developed a high-throughput genetic linkage strategy. Strains of interest were selected from the BY4741 (MATa *his3*Δ1, *leu2*Δ0, *ura3*Δ0, *met15*Δ0) haploid deletion set (Open Biosystems, Huntsville, AL, USA) and gridded in 96-well format. These strains were crossed to YAD641 (MAT*α can1*Δ:: *pMFA1-HIS3*, *his3Δ1*, *lys2Δ0*, *leu2Δ0*) by replica plating the grid to a YAD641 lawn and allowing cells to mate overnight on YPD at 30°C. Diploids were selected by growth on SD +histidine +leucine +G418 [66] for two days at 30°C, transferred to sporulation medium [66] by replica plating, and allowed to sporulate for 6 days at room temperature. Sporulated tetrads were then digested with 1 mg/ml zymolyase for 30 minutes at 37°C and deletion-containing MATa haploid progeny were selected by growth on SC -histidine -arginine +canavanine +G418 [66]. For each strain, 10 single colonies were chosen and re-screened for all phenotypes of interest. Strains with colonies that showed differences in growth rates on any of the media were dismissed as having unlinked mutations present in the strains.

### Flux balance analysis

FBA is a constraint-based approach for predicting steady state reaction rates (fluxes) in a metabolic network, and has been described in detail elsewhere [67]. FBA relies on the hypothesis that, on average, the concentrations of all metabolites in an asynchronous population of cells can be considered constant in time. This implies that the net sum of the fluxes producing and consuming any intracellular metabolite is zero. The network of reactions is uniquely defined by a stoichiometric matrix *S*, whose element *S*_*ij *_represents the stoichiometry of metabolite *i *in reaction *j*, positive if a metabolite is produced, negative if it is consumed. Exchange reactions, representing the fluxes of metabolites in and out of the system, are incorporated into the *S *matrix. The steady state constraint is hence formally expressed as:

*S*·*v *= 0

where *v *is the vector of reaction fluxes. In addition to the steady state assumption, inequality constraints can be imposed to set upper and lower bounds on individual fluxes (*α*_*i *_≤ *v*_*i *_≤ *β*_*i*_). As done before, we use these constraints to impose maintenance requirements and irreversibility of specific reactions, and to set limits on nutrient uptake rates. See Table S4 in Additional data file 2 for bounds used to implement each of the modeled conditions.

In the second step of FBA, linear programming is used to identify, among the flux vectors that satisfy the above constraints, one that optimizes a given objective function. In the current FBA calculations we used as our objective function the biomass flux (*v*_*growth*_), corresponding to a requirement of optimal utilization of resources towards maximal growth. The linear programming problem solved is therefore:

$$\begin{array}{ll} \max & v_{growth}\\ s.t. & S\cdot v=0\\ & \alpha_{i}\leq v_{i}\leq \beta_{i} \end{array}$$

Previous work has shown that when working with genome scale metabolic models, the above linear program can have multiple solutions [29,56]. In other words, for a given set of constraints, there can be several different sets of fluxes that result in an optimal value for *v*_*growth*_. For predicting growth rates using FBA the alternative optima are not a problem, but for establishing wild-type fluxes to use with MOMA and for analyzing specific fluxes underlying model predictions, it was important that we did not arbitrarily select among the multiple optimal solutions. To address this issue we performed a secondary optimization in which we selected, among all flux vectors that had the maximal value for *v*_*growth*_, the one that had the minimum sum of absolute values of fluxes. The motivation for this secondary optimization is that an organism may attempt to maximize growth with a minimum investment of resources. We utilized the commercial software Xpress (Dash Optimization, Englewood Cliffs, New Jersey, USA) to carry out all linear optimizations, as well as quadratic optimizations (see below).

In order to implement the deletion of a gene *g*, additional constraints are added, which set the flux through all reactions requiring the protein product of gene *g *to zero. Before comparing model predictions of mutant growth to the discretized experimental growth rates (see above), model growth rates were first normalized to be between 0 and 2. This allowed for a direct comparison of experimental growth rates and model predictions. If the difference between the mean experimental growth rate for two duplicates and the normalized model growth rate was less than or equal to one, then the model prediction was deemed correct. For comparison between FBA and MOMA, this threshold was lowered to 0.9, in order to identify more differences between the two optimization approaches.

### Minimization of metabolic adjustment

In addition to determining mutant phenotypes using FBA, we also utilized the minimization of metabolic adjustment (MOMA) approach [29]. Rather than making the unrealistic assumption of optimal growth for a mutant strain, MOMA hypothesizes that the flux distribution for the mutant will be minimally distant from the wild-type flux distribution. This is implemented using the following quadratic programming calculation:

$$\begin{array}{ll} \min & \sum_{i}{\left(v_{i}^{WT}-v_{i}^{\Delta X}\right)}^{2}\\ s.t. & S\cdot v=0\\ & \alpha_{i}\leq v_{i}\leq \beta_{i} \end{array}$$

### Yeast models

Yeast models were retrieved either from authors' websites or supplementary materials [11,13,25,68]. Subsequent to retrieving the models, some adjustments were made to each to allow for mimicking of the experimental media conditions (Table S5 in Additional data file 2). In particular, modifications were made to each model to allow for raffinose metabolism, as all models were lacking raffinose transporters. In addition, to mimic the experimental strains used in this study, the reactions encoded by His3, Leu2 and Ura3 were constrained to have zero flux.

### Flux visualization using VisANT

VisANT is a Java-based, platform-independent tool for the visualization and analysis of biological networks and is freely available for download over the web at [69]. VisANT provides a platform for analyzing diverse types of biological data through integration with many popular biological databases (for example, GenBank, SwissProt, and KEGG) and is capable of performing complex calculations of specific network properties. Metabolic networks were represented in VisANT as bipartite graphs, with the two classes of nodes being reactions and metabolites. Edges can either go into or come out of a reaction depending on whether the connected metabolite is a reactant or product, respectively. The weight and corresponding thickness of an edge between a metabolite and a reaction is representative of the normalized flux carried by the given reaction. All fluxes are normalized by the maximal flux in a given network, such that the maximum weight is unity.

In order to standardize the layouts of the network graphs, such that they more closely resembled traditional textbook representations of the corresponding biochemical pathways, we extracted the node coordinates from a template network. This was done by parsing the VisML file associated with the template network. VisML is an XML formatted representation that contains the necessary information to reconstruct a VisANT network. Once the node coordinates were extracted, different flux distributions could be overlaid on the same network map. As a supplement to this paper we have provided an online interface through which the flux predictions made by the iLL672 and iFF708 models, for all single deletion mutants, can be visualized in VisANT [50].

## Abbreviations

ETC: electron transport chain; FAD: flavin adenine dinucleotide; FBA: flux balance analysis; MOMA: minimization of metabolic adjustment; SD: standard deviation.

## Authors' contributions

AMD, GMC and DS conceived of the study. AMD designed the experiments. AMD, DJ and KW performed the experiments. ESS and DS designed and performed the computational analyses. ESS, AMD and DS wrote the manuscript.

## Additional data files

The following additional data files are available with the online version of this paper. Additional data file 1 includes supplementary Figures S1 and S2, along with the corresponding legends. Additional data file 2 contains supplementary Tables S1-S8.

## Supplementary Material

Additional data file 1 Figure S1a-c are heatmaps representing the concordance between the experimental gene deletion phenotypes and the corresponding predictions made by each of the three yeast models. Figure S2a, b are comparisons of the experimental data generated in the current study with similar data generated in previous studies using different experimental approaches. Click here for file

Additional data file 2 Table S1 contains the original experimental phenotype measurements for 465 gene deletion strains under the 16 different conditions. Table S2 contains the changes made to the experimental data based on the re-checking of mutants whose original phenotype disagreed with iFF708 model predictions. Table S3 contains the refined experimental data. Table S4 contains the upper bounds used for the three yeast models in order to model the 16 experimental conditions. Table S5 contains a list of changes made to the three models used in the current study. Table S6 contains a list of mutants that were predicted to be discordant by the iFF708 model, and therefore submitted to experimental verification. Table S7 contains guidelines for interpreting patterns of discordance between experimentally generated mutant phenotypes and corresponding model predictions. Table S8 contains a list of all discordances that remained after the experimental refinement, and our interpretation of them, based on the guidelines laid out in Table S7. Click here for file
