# Supplementary material for: Model-driven analysis of experimentally determined growth phenotypes for 465 yeast gene deletion mutants under 16 different conditions
Source: Genome Biol. 2008 Sep 22;9(9):R140. doi: 10.1186/gb-2008-9-9-r140 (PMC2592718; doi:10.1186/gb-2008-9-9-r140)
Supplement: Additional data file 1 — Figure S1a-c are heatmaps representing the concordance between the experimental gene deletion phenotypes and the corresponding predictions made by each of the three yeast models. Figure S2a, b are comparisons of the experimental data generated in the current study with similar data generated in previous studies using different experimental approaches. [file gb-2008-9-9-r140-S1.pdf]

Supplemental Figure Descriptions

Figure S1- Concordance maps for predictions made by 3 yeast models against experimentally determined essentiality

Concordance maps were constructed displaying the accuracy of predictions made by the (A) iFF708, (B) iLL672 and (C) iND750 models. The columns of each map represents the 16 conditions under which deletion viabilities were determined and the rows are deletion mutants for which there was at least one discordant prediction. Because each model had different predictions, and therefore different discordances with the experimental data, each model's concordance map has a different set of genes. Concordance patterns for predictions made using both FBA and MOMA are incorporated into the maps. Different colors indicate different combinations of experimental, FBA and MOMA predictions. White and gray boxes represent experimental viabilities and essentialities that were correctly predicted using both FBA and MOMA. Light and dark blue boxes represent experimental viabilities and essentialities that were incorrectly predicted using both FBA and MOMA. Dark red boxes represent experimentally viable genes correctly predicted by FBA, and not MOMA. Light red boxes represent experimentally essential genes correctly predicted by MOMA, and not by FBA.

Figure S2 – Comparison of experimental phenotypes to previously published data sets

Comparisons of the phenotype data generated in the current study were performed with previously published data sets from (A) Steinmetz et al., Nature Genetics, 2002 and (B) Kuepfer et al., Genome Research, 2005. (A) The data from Steinmetz et al. was generated using the commonly applied competitive batch assay. There were four conditions which overlapped between the Steinmetz data and the current data set (YPD, YPGly, YPEtOH and YPLac). While there is a strong correlation between our data and the Steinmetz data based on the illustrated boxplots, there are some outliers. A potentially interesting question is whether these differences are because of experimental artefacts of one of the two assays, or if they represent a set of genes which have differential importance in a competitive vs. non-competitive environment. (B) The data generated by Kuepfer et al. was done using a plate assay. Therefore the strong similarity between the two data sets is not surprising. It was difficult to do a comprehensive comparison between the two methods, as there was only an overlap in a single condition (minimal glucose media) between the two data sets.

Figure S1A

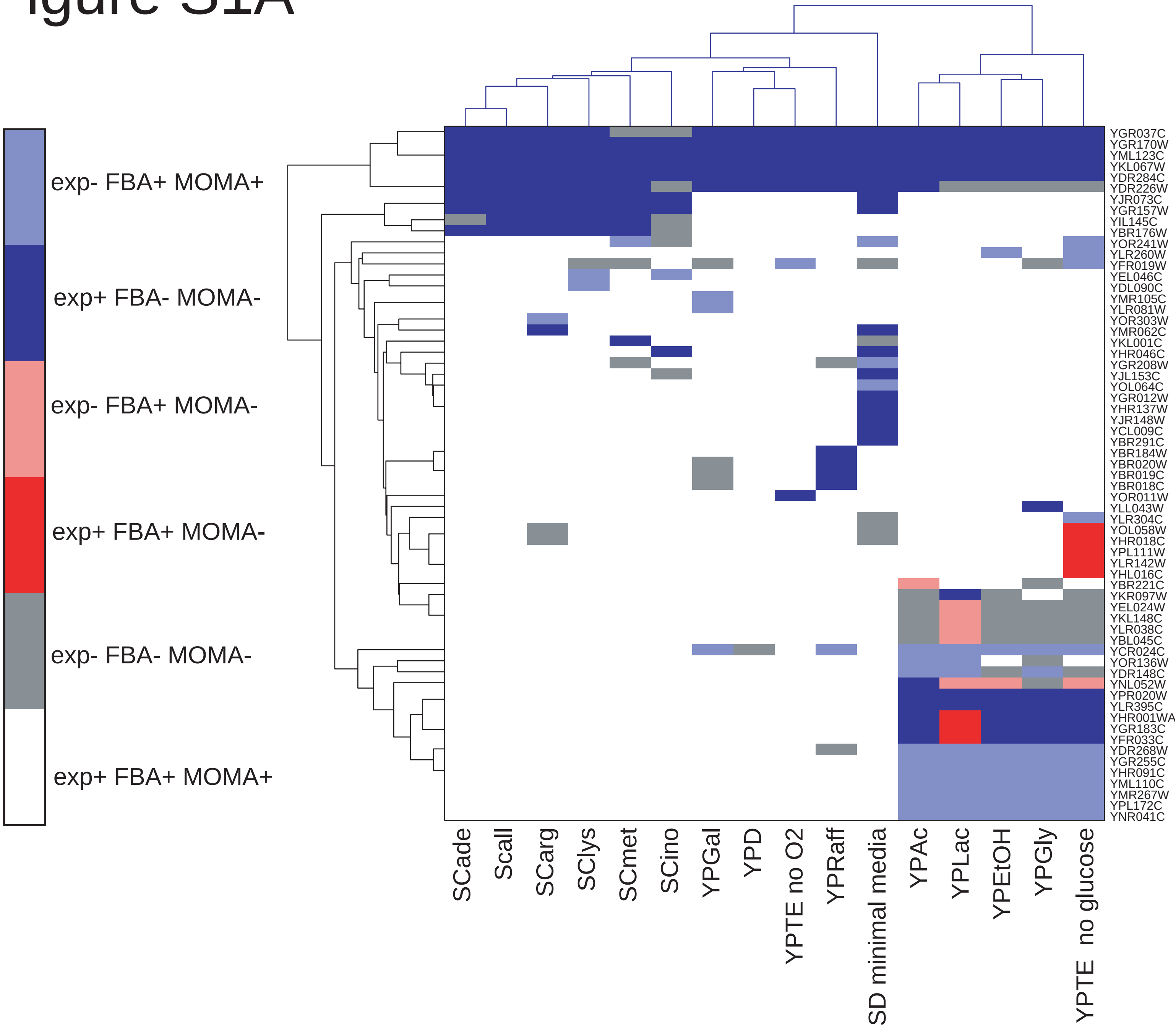
exp- FBA+ MOMA+
exp+ FBA- MOMA-
exp- FBA+ MOMA-
exp+ FBA+ MOMA-
exp- FBA- MOMA-
exp+ FBA+ MOMA+
SCade
Scall
SCarg
SClys
SCmet
SCino
YPGal
YPD
YPTE no O2
YPRaff
SD minimal media
YPAc
YPLac
YPEtOH
YPGly
YPTE no glucose
YGR037C
YGR170W
YML123C
YKL067W
YDR284C
YDR226W
YJR073C
YGR157W
YIL145C
YBR176W
YOR241W
YLR260W
YFR019W
YEL046C
YDL090C
YMR105C
YLR081W
YOR303W
YMR062C
YKL001C
YHR046C
YGR208W
YJL153C
YOL064C
YGR012W
YHR137W
YJR148W
YCL009C
YBR291C
YBR184W
YBR020W
YBR019C
YBR018C
YOR011W
YLL043W
YLR304C
YOL058W
YHR018C
YPL111W
YLR142W
YHL016C
YBR221C
YKR097W
YEL024W
YKL148C
YLR038C
YBL045C
YCR024C
YOR136W
YDR148C
YNL052W
YPR020W
YLR395C
YHR001WA
YGR183C
YFR033C
YDR268W
YGR255C
YHR091C
YML110C
YMR267W
YPL172C
YNR041C

Figure S1B

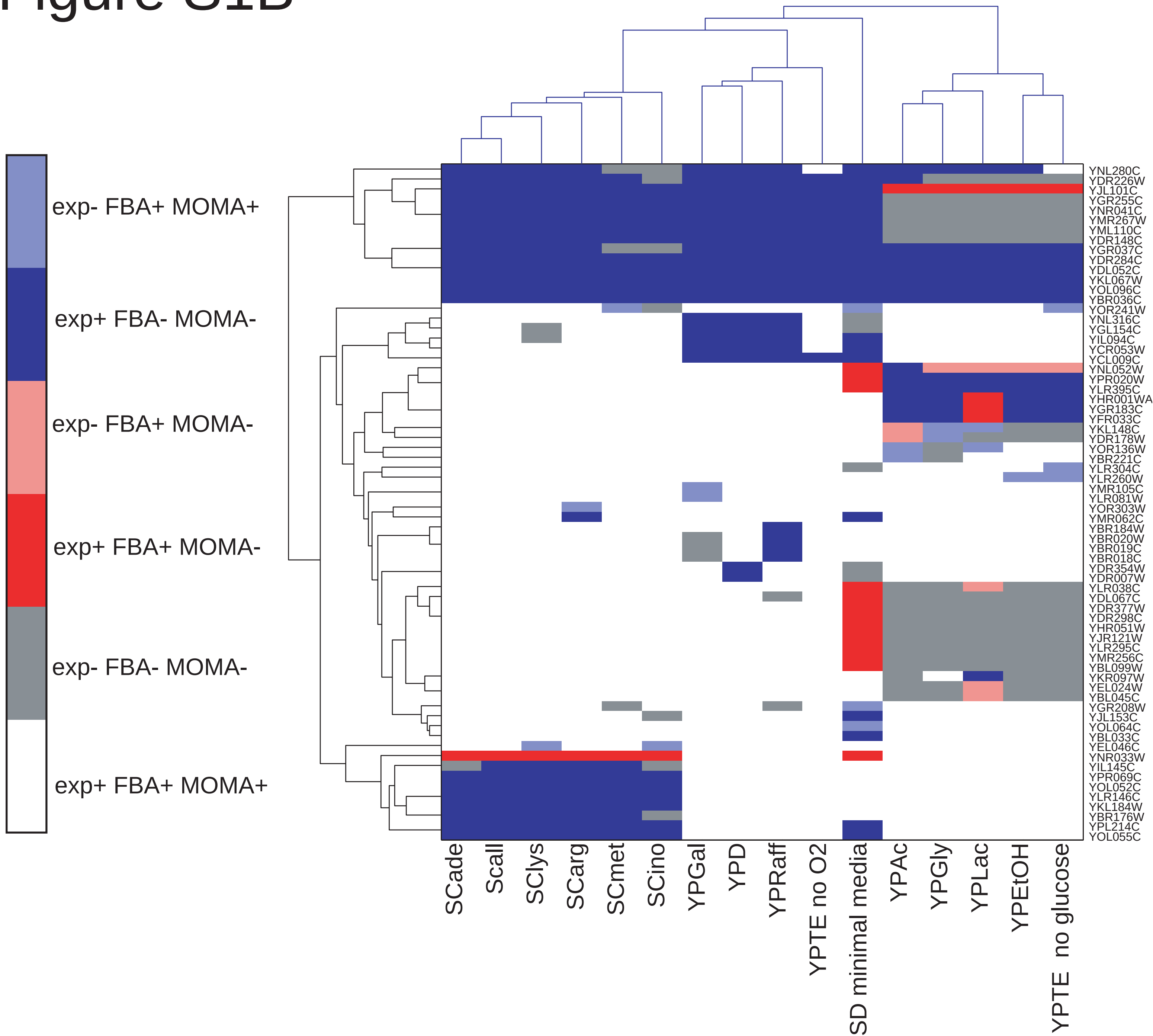

exp- FBA+ MOMA+
exp+ FBA- MOMA-
exp- FBA+ MOMA-
exp+ FBA+ MOMA-
exp- FBA- MOMA-
exp+ FBA+ MOMA+
YNL280C
YDR226W
YJL101C
YGR255C
YNR041C
YMR267W
YML110C
YDR148C
YGR037C
YDR284C
YDL052C
YKL067W
YOL096C
YBR036C
YOR241W
YNL316C
YGL154C
YIL094C
YCR053W
YCL009C
YNL052W
YPR020W
YLR395C
YHR001WA
YGR183C
YFR033C
YKL148C
YDR178W
YOR136W
YBR221C
YLR304C
YLR260W
YMR105C
YLR081W
YOR303W
YMR062C
YBR184W
YBR020W
YBR019C
YBR018C
YDR354W
YDR007W
YLR038C
YDL067C
YDR377W
YDR298C
YHR051W
YJR121W
YLR295C
YMR256C
YBL099W
YKR097W
YEL024W
YBL045C
YGR208W
YJL153C
YOL064C
YBL033C
YEL046C
YNR033W
YIL145C
YPR069C
YOL052C
YLR146C
YKL184W
YBR176W
YPL214C
YOL055C
SCade
Scall
SClys
SCarg
SCmet
SCino
YPGal
YPD
YPRaff
YPTE no O2
SD minimal media
YPAc
YPGly
YPLac
YPEtOH
YPTE no glucose

Figure S1C

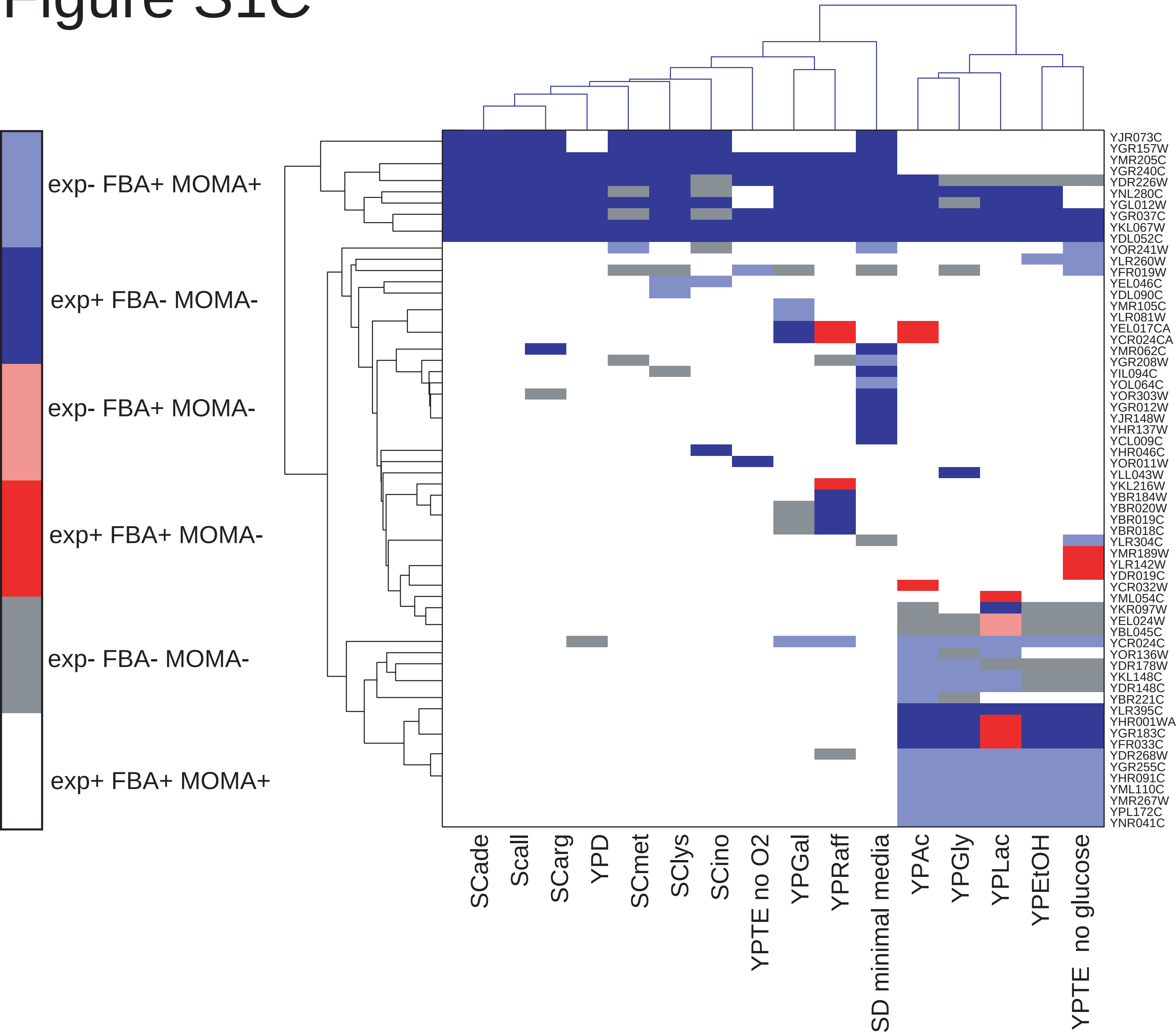

exp- FBA+ MOMA+
exp+ FBA- MOMA-
exp- FBA+ MOMA-
exp+ FBA+ MOMA-
exp- FBA- MOMA-
exp+ FBA+ MOMA+
YJR073C
YGR157W
YMR205C
YGR240C
YDR226W
YNL280C
YGL012W
YGR037C
YKL067W
YDL052C
YOR241W
YLR260W
YFR019W
YEL046C
YDL090C
YMR105C
YLR081W
YEL017CA
YCR024CA
YMR062C
YGR208W
YIL094C
YOL064C
YOR303W
YGR012W
YJR148W
YHR137W
YCL009C
YHR046C
YOR011W
YLL043W
YKL216W
YBR184W
YBR020W
YBR019C
YBR018C
YLR304C
YMR189W
YLR142W
YDR019C
YCR032W
YML054C
YKR097W
YEL024W
YBL045C
YCR024C
YOR136W
YDR178W
YKL148C
YDR148C
YBR221C
YLR395C
YHR001WA
YGR183C
YFR033C
YDR268W
YGR255C
YHR091C
YML110C
YMR267W
YPL172C
YNR041C
SCade
Scall
SCarg
YPD
SCmet
SClys
SCino
YPTE no O2
YPGal
YPRaff
SD minimal media
YPAc
YPGly
YPLac
YPEtOH
YPTE no glucose

Figure S2A

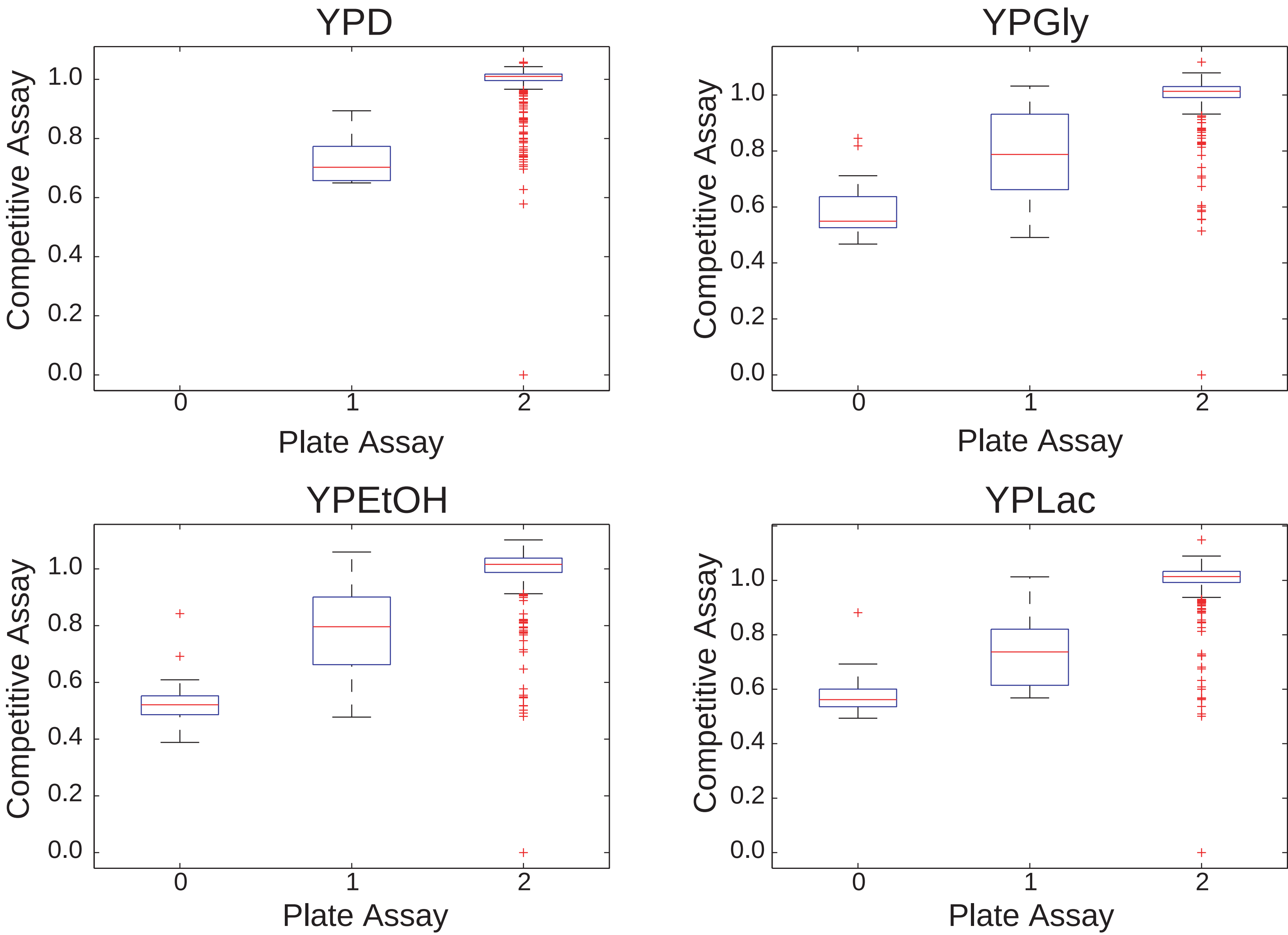
YPD
YPGly
YPEtOH
YPLac
Competitive Assay
Plate Assay
0.0
0.2
0.4
0.6
0.8
1.0
0
1
2

Figure S2B

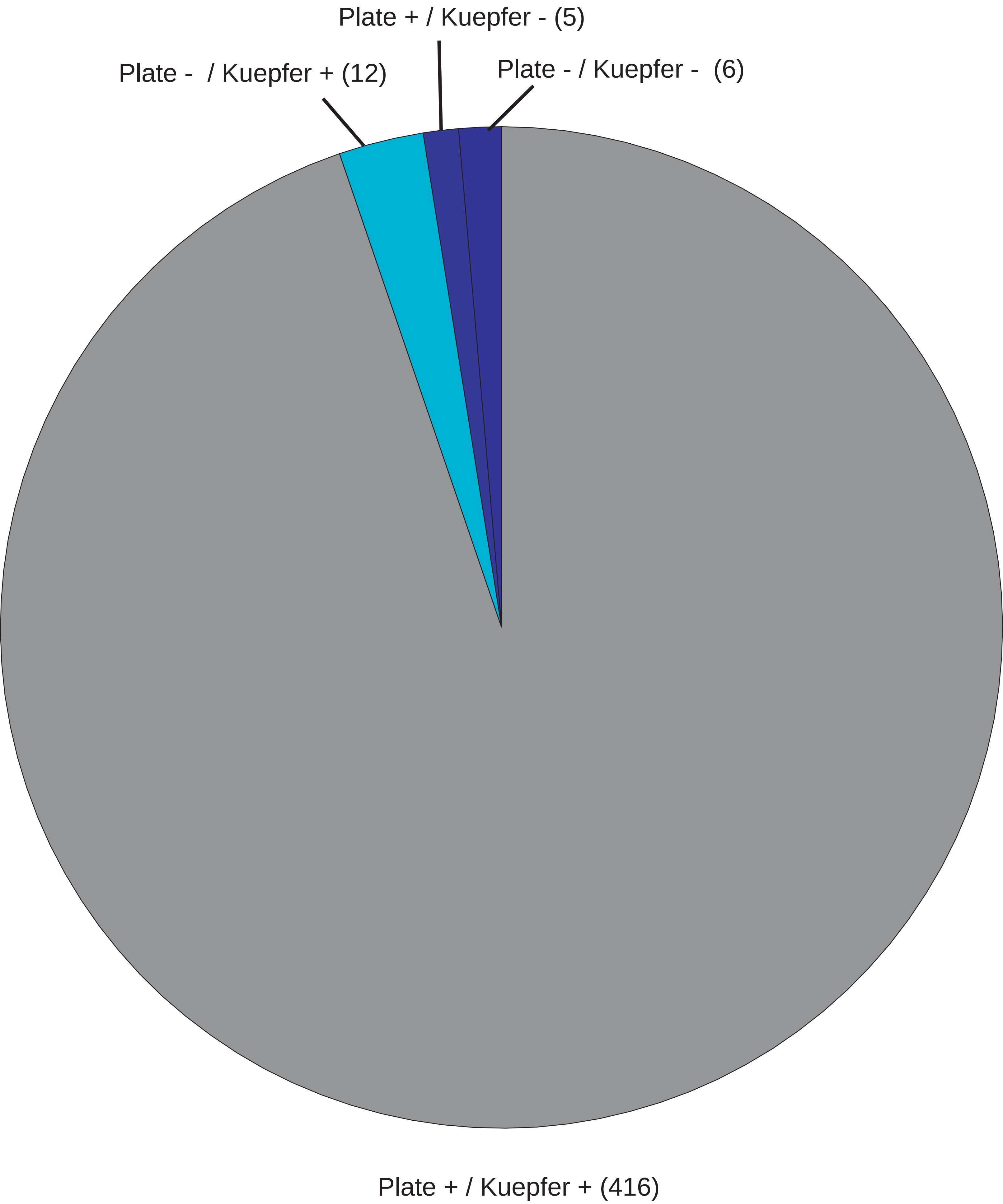
Plate + / Kuepfer - (5)
Plate - / Kuepfer + (12)
Plate - / Kuepfer - (6)
Plate + / Kuepfer + (416)
